# A Method for Tooth Model Reconstruction Based on Integration of Multimodal Images

**DOI:** 10.1155/2018/4950131

**Published:** 2018-06-20

**Authors:** Xinwen Zhou, Yangzhou Gan, Jing Xiong, Dongxia Zhang, Qunfei Zhao, Zeyang Xia

**Affiliations:** ^1^Department of Automation, Shanghai Jiao Tong University, Shanghai 200240, China; ^2^Shenzhen Institutes of Advanced Technology, Chinese Academy of Sciences, Shenzhen 518055, China; ^3^CAS Key Laboratory of Human-Machine Intelligence-Synergy Systems, Shenzhen Institutes of Advanced Technology, Shenzhen 518055, China

## Abstract

A complete digital tooth model is needed for computer-aided orthodontic treatment. However, current methods mainly use computed tomography (CT) images to reconstruct the tooth model which may require multiple CT scans during orthodontic progress, and the reconstructed model is also inaccurate in crown area. This study developed a tooth model reconstruction method based on integration of CT images and laser scan images to overcome these disadvantages. In the method, crown models and complete tooth models are first reconstructed, respectively, from laser scan images and CT images. Then, crown models from laser scan images and tooth models from CT images are registered. Finally, the crown from laser scan images and root from CT images were fused to obtain a new tooth model. Experimental results verified that the developed method is effective to generate the complete tooth model by integrating CT images and laser scan images. Using the proposed method, the reconstructed models provide more accurate crown than CT images, and it is feasible to obtain complete tooth models at any stage of orthodontic treatment by using one CT scan at the pretreatment stage and one laser scan at that stage to avoid multiple CT scans.

## 1. Introduction

In clinical orthodontics, a digital three-dimensional (3D) complete tooth model is needed for diagnosis, treatment planning, appliance design, tooth movement monitoring, and so on. With the development of the imaging and computer techniques, it is feasible to reconstruct digital tooth models from 3D dental images to conduct computer-aided orthodontic treatment.

Currently, laser scan images and computed tomography (CT) images are the most widely used 3D images in clinical orthodontics. Laser scan images have a high resolution up to ten-micron level, and the reconstructed tooth models have been used in space analysis, diagnosis, and computer-aided design of personalized orthodontic appliance, and so on [1–3]. However, the laser scan images only provide the 3D information of crown surface, and CT images is necessary in these applications where 3D information of root is needed including tooth arrangement, tooth movement monitoring, and orthodontic treatment simulation [4–7]. Complete 3D tooth models can be reconstructed from CT images [8, 9]. However, the reconstructed model from CT images is inaccurate in the crown area to be employed for personalized orthodontic appliance design due to the low image resolution [10]. In addition, multiple CT scans are needed in clinic for progress and posttreatment records, which is not recommended, since the subject would be exposed to high level of radiation [10, 11].

In this study, a new tooth model reconstruction method based on integration of laser scan images and CT images was developed. Crown models and complete tooth models were first reconstructed, respectively, from laser scan images and CT images. The crown models and tooth models were then registered to align the crown part of the two types of models. New complete tooth models were finally generated by fusing the crown from laser scan images and root from CT images. The fusion of laser scan images and CT images is challenging. It is difficult to extract the virtual root boundary (the boundary to be combined with the crown from laser scan images) from the CT tooth model because of the complex boundary of laser crown. Additionally, it is difficult to combine the registered crown and root models since the density of vertexes of the two models is inconsistent due to different image spatial resolutions of two sources.

Compared to the previous tooth model reconstruction method from laser scan images or CT images, the contribution of this study mainly includes two aspects. First, the reconstructed tooth model using the developed method can provide the complete 3D information of tooth, and the crown part is accurate enough to be applied for appliance design. Second, by applying the developed method, it is feasible to obtain the complete tooth model at any stage of orthodontic treatment by using one CT scan at pretreatment stage and one laser scan at that stage to avoid multiple CT scans.

## 2. Methods

**Figure 1:**
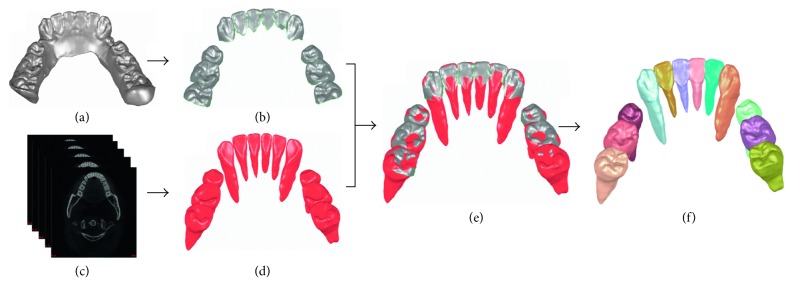
Framework of the tooth model reconstruction method based on integration of laser scan images and CT images. (a) Laser scan images of mandibular; (b) individual crowns segmented from laser scan images; (c) CT images of mandibular; (d) 3D tooth models reconstructed from CT images; (e) registration of crown models from laser scan images and CT images; (f) tooth models reconstruction by fusing crowns from the laser scan images and roots from CT images.

The framework of the tooth model reconstruction method based on integration of laser scan images and CT images is shown in Figure 1. The laser crown models (Figure 1) and completed CT tooth models (Figure 1) are firstly segmented and reconstructed from laser scan images (Figure 1) and CT images (Figure 1), respectively. The two types of models are then registered (Figure 1) using principal component analysis (PCA) algorithm [12] and iterative closest point (ICP) algorithm [13] to align their crown parts. Finally, the crowns from laser scan images and roots from CT images are fused to generate new tooth models (Figure 1) using the Delaunay-based region-growing method [14].

### 2.1. Crown Model Segmentation from Laser Scan Images

**Figure 2:**
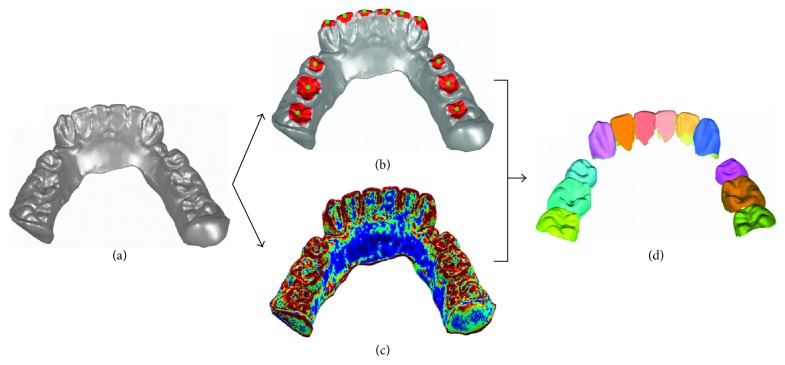
Modified fast watershed mesh segmentation method for individual crown model segmentation. (a) Mesh model from laser scanning of mandibular; (b) manually selected seed points of each crown (green points) and presegmentation result in the occlusion area of each crown; (c) height function distribution of facets; (d) individual crown segmented using the modified fast watershed mesh segmentation method.

The triangular mesh model (Figure 2) including all tooth crown surface in stereolithographic format is automatically obtained from the laser scanner. Individual tooth crown models (Figure 2) are segmented from the mesh model by applying a modified fast watershed mesh segmentation method.

In the commonly used fast watershed mesh segmentation method [15], a height function is defined based on the curvature of triangular facet to extract the boundary of neighboring mesh models. However, over segmentation may occur when using this method, and the method may fail to segment individual crowns due to the slow curvature change at the boundary between neighboring crowns. In this study, a modified fast watershed mesh segmentation algorithm is developed to segment individual crowns. Compared to the commonly used fast watershed mesh segmentation method, the modification mainly includes the following two points. (1) A region-growing algorithm [16] is applied to presegment the occlusion area of each crown to avoid over segmentation of the commonly used fast watershed mesh segmentation method (Figure 2). (2) Both curvature and area of triangular facet are employed to define a height function to extract boundary of neighboring crowns (Figure 2). For a given triangular facet *t*_1_, the height function *H*(*t*_1_, *t*_2_) between *t*_1_ and one of its first-order neighboring triangular facets *t*_2_ is written as

(1) $$\begin{array}{c} H\left(t_{1},t_{2}\right)=w\frac{\mathrm{area}\left(t_{1}\right)+\mathrm{area}\left(t_{2}\right)}{\max\left(\mathrm{area}\left(t_{i}\right)\right)}\;+\;\left(1-w\right)C\left(t_{1},t_{2}\right), \end{array}$$

where *t*_*i*_ represents the 1-neighboring facets of *t*_1_, area(·) is the area of a facet, *C*(*t*_1_, *t*_2_) is the curvature of *t*_1_ and *t*_2_ [15], and *w* is the weight factor which could be adjusted according to the model. In this study, *w* is empirically set to be 0.16.

### 2.2. Tooth Segmentation and Reconstruction from CT Images

**Figure 3:**
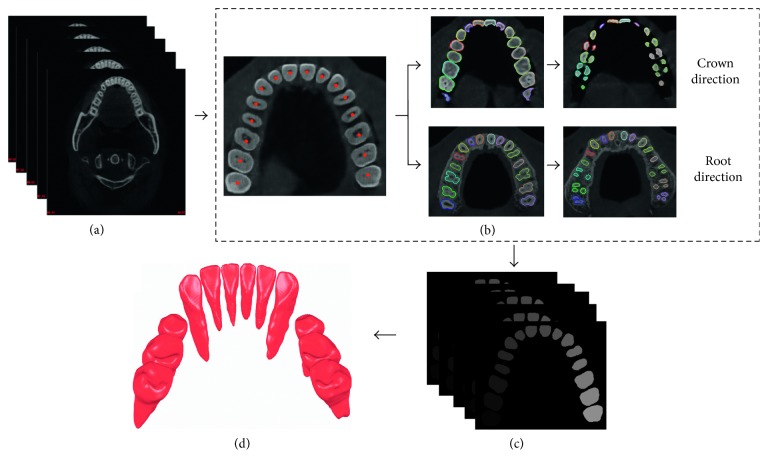
Framework of tooth segmentation and reconstruction from CT images. (a) CT images; (b) tooth contour segmentation slice-by-slice; (c) segmented individual tooth region in each slice; (d) reconstructed tooth models from the segmented tooth region using marching cube algorithm.

To reconstruct the complete tooth model from CT images (Figure 3), tooth contours (Figure 3) are first segmented from transverse section slice-by-slice (Figure 3) using the hybrid level set-based method [8, 9], and 3D tooth surface model (Figure 3) is reconstructed from the segmented tooth contours using the Marching Cube algorithm [17]. In the procedure of tooth contour segmentation (Figure 3), a user first manually selects a starting slice from the crown part of the volumetric CT images and picks seed points for each tooth in this slice. Then, the tooth contour is segmented slice-by-slice automatically from the CT images. The automatic segmentation starts from the selected starting slice and propagates along the crown and root directions for crown and root segmentation, respectively. For each slice, the Radon transform is employed to extract a separation line of neighboring teeth [9], and the hybrid level set model is then applied to segment each tooth contour from the mesial and distal sides of the corresponding separation line. Tooth contour propagation strategy which uses segmented tooth contour of previous slice as the tooth shape prior to current slice is employed to initialize the tooth contour automatically. More details of the hybrid level set-based tooth contour segmentation method can be found in [8].

### 2.3. Registration of Crown Models from CT Images and Laser Scan Images

In this study, the aim of model registration is to align the crown part of the two types of models. For the simplicity of computation, the tooth model reconstructed from CT images is sectioned using a plane to generate a crown model for registration with the crown model from laser scan images. The registration between the two types of models is performed through two steps: a coarse registration step based on PCA algorithm [12] and a fine registration step based on ICP algorithm [13]. During the registration process, the crown models from laser scan images are fixed, and the crown models from CT images are registered to the laser scan crown models.

In the coarse registration using PCA algorithm, the covariance matrix of each model is calculated, respectively, from the corresponding node coordinates. Three orthogonal unit eigenvectors are then extracted from the covariance matrixes, respectively, to establish the body-fitted coordinate system of the corresponding mesh model, and the origin of the coordinate system is set at the centroid of all the mesh model nodes. The aim of coarse registration is to find an affine transformation matrix such that the two body-fitted coordinate systems can be aligned after the affine transformation. Let CM_laser_ and CM_CT_ be the covariance matrixes of crown models from laser scan images and CT images, respectively, and EM_laser_ and EM_CT_ be the corresponding eigenvector matrixes. The rotation matrix *R*_1_ and the translation matrix *T*_1_ of the affine transformation can be obtained as follows:

(2) $$\begin{array}{c} R_{1}=EM_{\mathrm{laser}}\cdot \mathrm{Inv}\left(EM_{\mathrm{CT}}\right),\\ T_{1}=\mathrm{Centroid}\left(P_{\mathrm{laser}}\right)-R_{1}\cdot \mathrm{Centroid}\left(P_{\mathrm{CT}}\right), \end{array}$$

where Inv(·) is the matrix inverse operator, *P*_CT_ and *P*_laser_ are the node sets of the two mesh models, respectively, and Centroid(·) is the centroid of the node set operator. Then an affine transformation is performed on *P*_CT_ using *R*_1_ and *T*_1_, to generate the coarse registration result *P*_CCT_.

The fine registration using ICP algorithm aims at finding a set of affine transformation matrixes such that the mean square error (MSE) of the distance between the corresponding nodes of the laser scan crown and the coarsely registered CT crown models achieve minimum after the affine transformations. Let *M* and *N* denote the number of nodes in *P*_CCT_ and *P*_laser_, respectively. The procedure of the ICP algorithm for the fine registration is conducted as follows.

  Step 1. Build the *kd*-tree [18] of *P*_CCT_ and *P*_laser_, respectively.
  Step 2. Calculate the rotation matrix *R*_int_ and translation matrix *T*_int_ using the quaternion method [13].
  Step 3. Transform *P*_CCT_ using an affine transformation with parameters *R*_int_ and *T*_int_.
  Step 4. For each node *n*_*i*_(*i*=1,…, *M*) in *P*_CCT_, search its nearest node *n*_*j*_(*j*=1,…, *N*) in *P*_laser_ and calculate the corresponding Euclidean distance *D*_*ij*_ between *n*_*i*_ and *n*_*j*_. The registration error *E*_C_ is defined as

(3) $$\begin{array}{c} E_{C}=\sqrt{\frac{\sum_{i=1}^{M}D_{ij}^{2}}{M}}, \end{array}$$

  Step 5. Go back to step 2 until the registration error *E*_C_ is smaller than a preset threshold *E*_th_.

### 2.4. Fusion of Crown from Laser Scan Images and Root from CT Images

In this study, the Delaunay-based region-growing (DBRG) algorithm [14] is applied to generate a new tooth model by fusing the crown models from laser scan images and root models from CT images. In this procedure, each tooth model is proceeded independently using the DBRG algorithm. Two sets of triangle denoted by *F* and *R*_Q_, respectively, and a set of edges denoted by *E* are defined. The fusion of the laser scan crown and CT root models is performed as follows:

  Step 1. Calculate the Delaunay triangulation *T* of all the vertexes in the two registered models.
  Step 2. Choose a starting triangle from *T*, put the starting triangle into *F*, and put its edges into *E* (the starting triangle is selected from those triangles with a largest *z* coordinate of vertexes and minimum circumradius).
  Step 3. Calculate the local smooth degree (LSD) of these triangles in *T* who have edges in *E* and put these triangles into *R*_Q_ (the definition of LSD can be found in [14]).
  Step 4. Denote the triangle with the largest LSD in *R*_Q_ by *t*_0_ and check whether the local geometry and topology between *t*_0_ and triangles in *F* is correct. If it is correct, (a) remove *t*_0_ from *R*_Q_, (b) put *t*_0_ into *F*, (c) put edges of *t*_0_ into *E*, and (d) delete the edges in *E* that are no longer the boundary edges of *F*. If not, remove *t*_0_ from *R*_Q_ and repeat Step 4 until *R*_Q_ is empty.
  Step 5. Go back to Step 3 until *R*_Q_ is empty.

## 3. Experiments

This study was reviewed and approved by Institutional Review Board of Shenzhen Institutes of Advanced Technology, Chinese Academy of Sciences. Written informed consents of the subjects are obtained. Intraoral laser scan images and CBCT images of five subjects (4 male, 1 female; average age 18 ± 1.9) with crowded teeth in need of orthodontic treatment are obtained at the pretreatment stage. The laser scan images and CBCT images have isotropic voxel sizes of 50 *μ*m and 0.125 mm, respectively.

### 3.1. Results of Model Reconstruction from 3D Images

**Figure 4:**
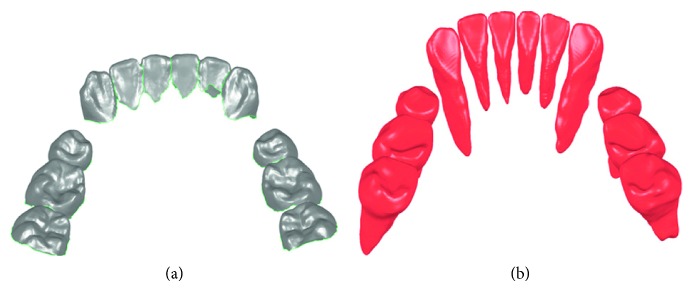
Crown and tooth model reconstruction results of one subject. (a) Crown models reconstructed from laser scan images; (b) tooth models reconstructed from CBCT images.

Tooth models of one subject reconstructed from laser scan images and CBCT images are shown in Figures 4 and 4, respectively. Visually, the crown models reconstructed from laser scan images provide more detail information of crown than those from CBCT images.

### 3.2. Results of Crown Model Registration

**Figure 5:**
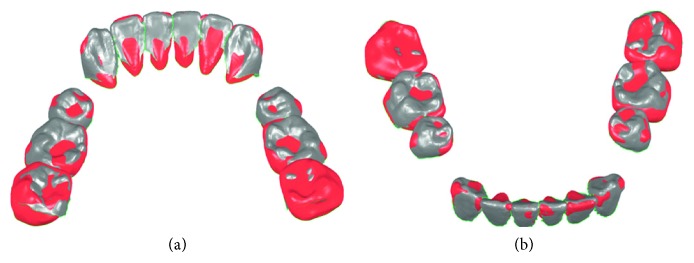
Crown registration results from lingual view (a) and labial view (b). Red: the crown model based on CT images; gray: the crown model based on laser scan images.

**Figure 6:**
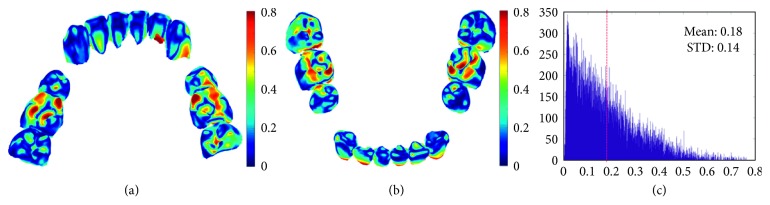
Error map of registration result from lingual view (a) and labial view (b), and the histogram of distance between the corresponding nodes of two model (c).

The superimposed crown models from laser scan images and CBCT images of one subject after model registration are shown in Figure 5, and the corresponding registration error distribution of nodes is shown in Figure 6.

In this study, the average distance (AD) from the laser scan crown model to the registered CT crown model is applied to quantify the registration error of the two models. The registration error for all the tested subjects is 0.19 ± 0.03 mm, and the registration error of each subject is listed in Table 1.

### 3.3. Results of Model Fusion

**Figure 7:**
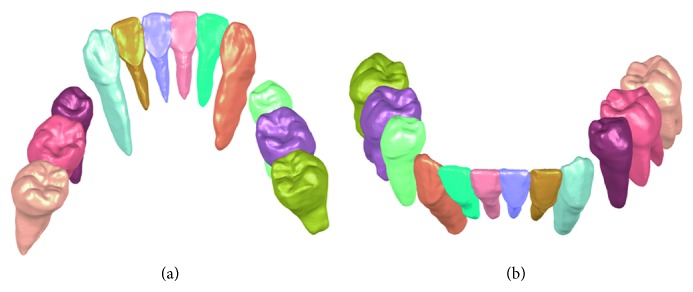
Tooth models from the fusion of crown from laser scan images and root from CT images. (a) Lingual view of fused tooth models; (b) labial view of fused tooth models.

For all the tested images, new tooth models are successfully generated based on the fusion of the crown from laser scan images and the root from CT images.  Figure 7 shows the generated tooth models of one subject based on the fusion. Compared to the models directly reconstructed from laser scan images and CT images in Figure 4, the models obtained by the fusion method not only contain the complete 3D tooth information but also provide accurate crown.

## 4. Discussion

In computer-aided orthodontics, complete 3D digital tooth models are needed for diagnosis, treatment planning, and treatment simulation. One can reconstruct the complete tooth models from CT images. However, the reconstructed models are inaccurate in the crown part for personalized appliance design. Additionally, multiple CT scans are necessary for progress and posttreatment records if only using the CT image to reconstruct the tooth models. Compared with CT images, laser scan images can be used to reconstruct much more accurate crown models without radiation [19, 20]. In this study, we developed a new tooth model reconstruction method based on the fusion of the crown model from laser scan images and root model from CT images. By applying the developed method, it was feasible to obtain the complete tooth model at any stage of orthodontic treatment by using one CT scan at pretreatment stage and one laser scan at that stage to avoid multiple CT scans. Additionally, the reconstructed tooth model using the developed method provided more accurate crown than CT images.

**Figure 8:**
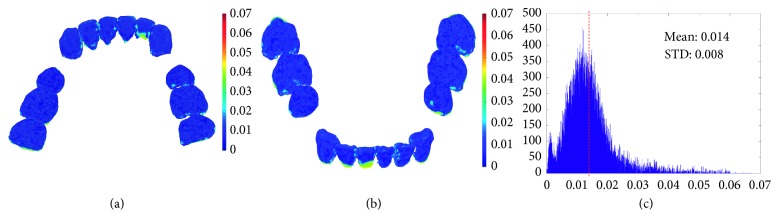
Error between the laser scan crown model and the tooth models from the fusion procedure. (a) Lingual view; (b) labial view; (c) histogram of the error.

In the developed method, the crown models from laser scan images and the tooth models from CT images are first registered and then fused to generate the complete tooth models, and both the registration and fusion procedure would affect the accuracy of the reconstructed model. In previous works of the dental model registration, the mean registration errors are among 0.1 mm and 0.3 mm [21–23]. In this study, the mean registration error in the developed method is 0.19 mm and is clinically acceptable [24]. To evaluate the model fusion error, the laser scan crown is chosen as the ground truth and compared with the fused tooth model, and AD was used to quantify the fusion error. The error map of one subject is shown in Figure 8. For all the tested subjects, the fusion error is 0.015 ± 0.004 mm which is rather small and clinically acceptable [25], and the fusion error of each subject is listed in Table 2.

In the developed method, manual initialization is needed for the segmentation of CT images and laser scan images which leaded to limited difference of segmentation results for different trained users [8, 15]. While the registration and fusion procedure are performed fully automatically, we could thus achieve results with good reproducibility and reliability compared to those manually operating works [21, 25]. The proposed method may fail to reconstruct angled teeth since the slice-by-slice image segmentation strategy used in the method has difficulty in the segmentation of these teeth [26].

## 5. Conclusion

This study presented a new tooth model reconstruction method based on integration of laser scan images and CBCT images. Compared to those commonly used tooth model reconstructed methods which directly reconstruct complete tooth model from CT images, the proposed method can generate tooth model with more accurate crown and can obtain a complete tooth model at any stage of orthodontic treatment by using one CT scan at pretreatment stage and one laser scan at that stage to avoid multiple CT scans. Thus, the proposed tooth model reconstruction method based on image integration can benefit the computer-aided orthodontic treatment.

## Figures and Tables

**Table 1:** Registration error of each subject.

Subjects	AD (mm)
Subject 1	0.18
Subject 2	0.16
Subject 3	0.23
Subject 4	0.21
Subject 5	0.17
All	0.19 ± 0.03

**Table 2:** Fusion error of each subject.

Subjects	AD (mm)
Subject 1	0.014
Subject 2	0.011
Subject 3	0.020
Subject 4	0.017
Subject 5	0.013
All	0.015 ± 0.004
